# Letter to the editor: Calcar fracture gapping: a reliable predictor of anteromedial cortical support failure after cephalomedullary nailing for pertrochanteric femur fractures

**DOI:** 10.1186/s12891-022-05688-w

**Published:** 2022-07-28

**Authors:** Akikazu Hagiyama, Norio Yamamoto, Yosuke Tomita, Koji Demiya

**Affiliations:** 1 Division of Physical Medicine and Rehabilitation, Okayama University Hospital, Okayama, Japan; 2 Scientific Research Works Peer Support Group (SRWS-PSG), Osaka, Japan; 3 Department of Orthopedic Surgery, Miyamoto Orthopedic Hospital, 4-2-63, Kunitomi, Naka-ku, Okayama, Okayama 773-8236 Japan; 4 Department of Physical Therapy, Faculty of Health Care, Takasaki University of Health and Welfare, Takasaki, Gunma Japan; 5 Department of Orthopedic Surgery, National Hospital Organization Shikoku Cancer Center, Matsuyama, Ehime Japan

**Keywords:** Anteromedial cortical support, Fracture gap, Trochanteric fracture, Multivariable analysis

## Abstract

A recently published article by Song H et al. investigated the risk factors for anteromedial cortical support loss in pertrochanteric fractures treated with cephalomedullary nails. In this Correspondence, we would like to raise some concerns. Specifically, calcar fracture gap and anteromedial cortical support are different concepts in evaluating reduction quality. In addition, calcar fracture gap using immediate postoperative radiographic images has measurement bias. Lastly, explanatory variables selected for multivariable analysis are inappropriate. We would like to discuss and suggest solutions for these problems.

## Main text

We read the article by Song H et al. [1] with great interest to investigate the risk factors for anteromedial cortical support loss in pertrochanteric fractures treated with cephalomedullary nails. However, we would like to raise some concerns.

1. Calcar fracture gap and anteromedial cortical support are different concepts in evaluating reduction quality using postoperative radiographic findings. Calcar fracture gap is the distance between proximal and distal fragments, while anteromedial cortical support is the relative positioning on bone contact between them [2, 3]. In radiographic findings, sometimes there is a calcar fracture gap when there is anteromedial cortical support. Conversely, there is no calcar fracture gap when there is anteromedial cortical support loss. In addition, the calcar fracture gap directly indicates anteromedial cortex-to-cortex support loss. Therefore, the estimation using the receiver operating characteristic curve is not suitable for them. We recommend that it is meaningful for surgeons to evaluate the association or difference between anteromedial cortical support using immediate postoperative plain radiographs and computed tomography (CT).

2. The primary outcome setting is not appropriate. We think that the primary outcome in this study is the anteromedial calcar support loss in postoperative CT, although it is defined as the degree of fracture gapping by the authors [1]. Appropriate selection of the primary outcome is essential to clarify the study objective.

3. The calcar fracture gap using immediate postoperative radiographic images has measurement bias. The three-dimensional or reconstruction images assessment using CT provides a more reliable measurement of the calcar fracture gap. The authors could use the postoperative CT images as reported in the study. However, we also understand that the study results obtained using plain radiographs would be more generalizable to clinical practice than the CT images. In order to increase the accuracy of the measurement using plain radiographs, the gap measurement can be calibrated to their actual values using a known length as a reference in the image, such as the length and diameter of the used implants (screw or nail) [4].

4. In the multivariable analysis, the selection of explanatory variables (reduction quality, calcar fracture gap [AP view and lateral view]) is inappropriate because these variables share similar measurement constructs. First, the criteria of reduction quality used in the study is a combined assessment method including the elements of medial and anterior cortex support [5], which are very similar to the anteromedial cortical support. Second, calcar fracture gap in both AP view and lateral view was included, although they may just be evaluating the same gap from different directions. Therefore, we are concerned with multicollinearity as a result of using explanatory variables that share similar measurement constructs. Furthermore, the choice of explanatory variables should not be based solely on a *p*-value (*p* < 0.10) in univariate analysis. As a rule of thumb, the explanatory variables will be selected based on the association with the objective variable. It is better to consider the assumption for multivariable logistic regression analysis [6]. We recommend removing reduction quality and calcar fracture gap from explanatory variables and selecting other explanatory variables, such as osteoporosis or surgeon’s experience.

Hence, these concerns highlight possible misinterpretations of the reported findings in the study by Song H et al. [1] and the potential influence of confounders that could be partly addressed by further study design considerations.

## Data Availability

Not applicable.

## Abbreviation

CT: Computed tomography
